# Exocrine Pancreatic Carcinogenesis and Autotaxin Expression

**DOI:** 10.1371/journal.pone.0043209

**Published:** 2012-08-29

**Authors:** Sandeep Kadekar, Ilona Silins, Anna Korhonen, Kristian Dreij, Lauy Al-Anati, Johan Högberg, Ulla Stenius

**Affiliations:** 1 Institute of Environmental Medicine, Karolinska Institutet, Stockholm, Sweden; 2 Computer Laboratory, University of Cambridge, Cambridge, United Kingdom; Northwestern University, United States of America

## Abstract

Exocrine pancreatic cancer is an aggressive disease with an exceptionally high mortality rate. Genetic analysis suggests a causative role for environmental factors, but consistent epidemiological support is scarce and no biomarkers for monitoring the effects of chemical pancreatic carcinogens are available. With the objective to identify common traits for chemicals inducing pancreatic tumors we studied the National Toxicology Program (NTP) bioassay database. We found that male rats were affected more often than female rats and identified eight chemicals that induced exocrine pancreatic tumors in males only. For a hypothesis generating process we used a text mining tool to analyse published literature for suggested mode of actions (MOA). The resulting MOA analysis suggested inflammatory responses as common feature. In cell studies we found that all the chemicals increased protein levels of the inflammatory protein autotaxin (ATX) in Panc-1, MIA PaCa-2 or Capan-2 cells. Induction of MMP-9 and increased invasive migration were also frequent effects, consistent with ATX activation. Testosterone has previously been implicated in pancreatic carcinogenesis and we found that it increased ATX levels. Our data show that ATX is a target for chemicals inducing pancreatic tumors in rats. Several lines of evidence implicate ATX and its product lysophosphatidic acid in human pancreatic cancer. Mechanisms of action may include stimulated invasive growth and metastasis. ATX may interact with hormones or onco- or suppressor-genes often deregulated in exocrine pancreatic cancer. Our data suggest that ATX is a target for chemicals promoting pancreatic tumor development.

## Introduction

Exocrine pancreatic cancer is the fifth major cause of cancer death in developed countries [1]. It is an aggressive tumor, characterized by invasive growth and early metastasis and the 5-year survival rate is 5% [2], [3]. Several studies show that men are more often affected than women [2], [4], [5], [6], [7]. Associated risk factors include cigarette smoking, environmental tobacco smoke [8], a diet high in fat and meat, obesity, diabetes mellitus, and consumption of soft drinks and juice [9], [10]. Recent pooled analyses suggest that high alcohol intake [11], or red meat consumption [12], are risk factors for pancreatic cancer among men but not among women. A similar response pattern has been reported for silica dust exposure [13]. An association with chronic pancreatitis has been observed for long [14] and a causative role of the cholecystokinin (CCK) analogue cerulein has been shown in mouse studies [15]. Acquired K-ras mutations are common (>95%) in pancreatic tumors [16]. Although there are germline mutations predisposing for pancreatic cancer, environmental factors are likely to induce somatic mutations that may be causative to the development of pancreatic cancer [1].

A role of environmental contaminants or xenobiotics has been studied to a limited extent. Animal experiments have shown that e.g. the antimetabolite azaserine can induce pancreatic tumors in both male and female rats [17], and studies of xenobiotics inducing exocrine pancreatic cancer have been reviewed [18]. Furthermore, the National Toxicology Program (NTP) database, which contains more than 477 reports on 2-year cancer bioassays employing male and female rats as test animals, shows that several chemicals increase the incidence of exocrine pancreatic tumors.

We have analysed gender differences in susceptibility to chemical carcinogens [19]. We studied the NTP database and found that among the chemicals tested in both sexes, ten induced exocrine pancreatic tumors in rats. Two of these affected both males and females, with a higher male incidence. Eight chemicals affected males only. This is in line with earlier observational [2], [7] and experimental [20] data, showing that the incidence of neoplasms of the exocrine pancreas in rats is higher in males than in females.

In order to understand intrinsic sex differences we investigated the eight chemicals that induced “male-specific” rat pancreatic tumors with the aim to find common mechanistic factors that could explain the male-specific effect. We found that these chemicals activate inflammatory response in human pancreatic cancer cells and that this was related to activation of ATX and MMP-9. We also provide evidence that these effects are associated with increased invasive growth.

## Materials and Methods

### Identification of chemicals causing exocrine pancreas tumors in rats

Data on bioassays of male and female rats were found on the webpage of National Toxicology Program (NTP) (http://ntp.niehs.nih.gov/). The NTP long-term toxicology and carcinogenesis studies (bioassays) in rodents generally employ both sexes of rats (Harlan Sprague Dawley) with three exposure concentrations plus untreated controls in groups of 50 animals for two years. In total, 17 chemicals or chemical mixtures were associated with exocrine pancreas tumor induction. Five chemicals (PCB 126, PCB 153, pentachlorodibenzofuran, PCB 118, TCDD and dioxin mixture) were excluded because they were only studied in females, one chemical (nitrofen) was excluded because the results in male rats were inconclusive; another chemical was excluded because evidence was not clear (2,2-bis(bromomethyl)-1,3-propanediol). Of the resulting ten chemicals eight induced tumors in male rats only, while two chemicals (butyl benzyl phthalate and dichlorvos) caused exocrine pancreatic tumors also in females. The eight remaining chemicals were 1,2,3-trichloropropane (TCP), 2-amino-5-nitrophenol (AMN), 2-mercaptobenzothiazole (MER), benzyl acetate (BA), chlorendic acid (ChlA), cinnamyl anthranilate (CA), roxarsone (ROX), 2,4-and 2,6-toluene diisocyanate (TDI), see Table 1. The majority of these chemicals are industrial chemicals. All eight were included in this study. In the NTP experiments, five chemicals (BA, CA, TCP, AMN, ROX) were given at the same doses per kg body weight for both male and female rats. In two bioassays, ChlA and TDI, female rats received a higher dose per kg body weight than males. One carcinogen, MER, was given at higher dose per kg body weight in male rats, since female rats gained less weight at higher doses.

**Table 1:** Chemicals classified by NTP to be associated with site-specific tumor induction in pancreas acinar cell (pancreatic acinar cell adenoma or carcinoma) in male rats and their major use.

Chemical	Use	Total PubMed abstracts	NTP Salmonella results
1,2,3-Trichloropropane	Paint and varnish remover, solvent and degreasing agent	65	+
2-Amino-5-nitrophenol	Colorant in hair dyes and used in manufacture of CI Solvent Red 8, azo dye	14	+
2-Mercaptobenzothiazole	Rubber accelerant and preservative	206	−
Benzyl acetate	Soap fragrance, flavoring ingredient	105	−
Chlorendic acid	Used in preparation of fire-retardant polyester resins and plasticizers	19	−
Cinnamyl anthranilate	A synthetic flavouring agent	25	−
Roxarsone	Veterinary drug used as a growth promoter and as an anticoccidial agent	126	−
2,4-Toluene-diisocyanate, 2,6-Toluene-diisocyanate	Used for manufacture of flexible polyurethane foams	1028	+

Total PubMed abstracts and NTP's Salmonella results are shown.

### Text mining-based analysis of published literature

We used a text mining tool for review and analysis of published literature for the eight rat male-specific tumor-inducing chemicals. The Cancer Risk Assessment and Biomedical (CRAB) text-mining tool displays, for a given chemical or group of chemicals, a publication profile, i.e. the distribution of PubMed abstracts over a taxonomy which specifies various types of scientific evidence for cancer risk assessment [21], [22], [23], [24]. The distributions were presented as the percentage of abstracts containing evidence for certain “mode of actions” (MOAs), i.e. key mechanistic events suggested being critical for cancer development for a given chemical. The MOA taxonomy captures the current understanding, often on a molecular level, of different processes leading to carcinogenesis [25]. In short, it divides two commonly used MOA types, genotoxic and non-genotoxic, into different subtypes following the classification of Hattis et al. [25]. The tool creates a publication profile for a chemical (or group of chemicals) by assigning each abstract to one or several MOA classes. It displays the results in (mean) % of the total number of abstracts for each chemical or group of chemicals. By comparing the publication profiles created by the tool shared properties of seemingly unrelated chemicals can be identified [21]. The distributions for the eight chemicals were compared with the distributions for six well-known genotoxic (benzo[*a*]pyrene, aflatoxin B1, 1,3-butadiene, 4-aminobiphenyl, *N*-ethyl-*N*-nitrosourea, 1,3-dichloropropene) and ten non-genotoxic compounds (TCDD, PCB126, 2,3,4,7,8-pentachlorodibenzofuran, fumonisin B1, bis(2-ethylhexyl)phthalate (DEHP), D-limonene, phenobarbital, tamoxifen, chloroform, diethylstilbestrol). These non-genotoxic compounds represent different sub-MOAs according to Hattis [25]. The text mining tool is available on request [21].

### Cell culture and reagents

Human pancreatic ductal cancer cell lines (Panc-1, MIA PaCa-2 and Capan-2), were obtained from the American Type Culture Collection, ATCC (Manassas, VA, USA). Dulbecco's modified Eagle's medium was supplemented with 10% calf serum and penicillin-streptomycin (0.1% serum for 48 hours for starvation). Panc-1 and MIA PaCa-2 cells originate from primary ductal tumors of male subjects and were derived from metastasized tumors with poor invasive capability, while Capan-2 is well- to moderately well-differentiated cell line [26]. Cells were treated with the following chemicals BA, ChlA, CA, TDI, TCP, AMN and MER (all from AlfaAesar, Germany), ROX, Fura-2 AM, testosterone and KN62 (from Sigma, Germany) and HA130 (Tocris bioscience). ChlA, AMN, MER and ROX were dissolved in acetone, BA, CA, TDI in DMSO and TCP in water. The final concentration of acetone or DMSO was <0.2%. All experiments were repeated at least three times with different batches of cells.

### Intracellular Ca^2+^measurement

Cells were incubated for 30 min at 37°C with 5 µM Fura-2AM. Fura-2AM is cleaved by intracellular esterases to form Fura-2, which subsequently binds to free Ca*^2+^*. This results in increased fluorescence of Fura-2. Unloaded Fura-2AM was removed by centrifugation at 150×*g* for 3 min. Cells were suspended in Krebs–Ringer buffer containing 125 mM NaCl, 5 mM KCl, 1.3 mM CaCl_2_, 1.2 mM KH_2_PO_4_, 1.2 mM MgSO_4_, 5 mM NaHCO_3_, 25 mM Hepes, 6 mM glucose, and 2.5 mM probenecid (pH 7.4). Fura-2AM-loaded cells were maintained at 25°C for 90 min before fluorescence measurement. The absorbance was measured at 340 nm.

### Western blotting

Cells were washed with PBS and lysed in IPB-7 containing protease inhibitors. Conditioned media was prepared by removing floating cells by centrifugation and thereafter the media was concentrated by using Amicon Ultra-50K filters. The samples were subjected to SDS-PAGE and thereafter blotted onto a polyvinylidene difluoride membrane (Bio-Rad, Hercules, CA). The protein bands were subsequently probed using antibodies against Cdk2, autotaxin, MMP-9 (Santa Cruz Biotechnology Santa Cruz, CA) or α-calcineurin (Sigma, Germany). Proteins were visualized using an enhanced chemiluminiscence procedure (Amersham Biosciences, Uppsala, Sweden). The Western blot results were analysed with NIH Image 1.62 software. All experiments were repeated at least three times with different batches of cells.

### RNA purification and Real-Time RT-PCR

Total cellular RNA was prepared using the RNeasy Mini Kit (Qiagen) and further treated with TURBO DNA-*free*™ (Ambion). cDNA was generated using the High Capacity cDNA Reverse Transcription kit (Applied Biosystems) according to protocol. Subsequently, quantification of gene expression was performed in duplicates using HotStart-IT® SYBR® Green qPCR Master Mix (USB) with detection on an Applied Biosystems 7500 Real-Time PCR System (Applied Biosystems). The primer sequences are shown in Table 2. Relative gene expression quantification was based on the comparative threshold cycle method (2^−ΔΔCt^) with normalization of the raw data to the included housekeeping gene (GAPDH).

**Table 2:** Primer sequences used for RT^2^-PCR.

Gene	Sequence
ENPP2	F: 5′-TATGCTGCGGAAACTCGTCAGG-3′
	R: 5′-GACGTTGACACACCGATGCAGT-3′
IL-8	F: 5′-GTGCAGTTTTGCCAAGGAGT-3′
	R: 5′-CTCTGCACCCAGTTTTCCTT-3′
MMP-9	F: 5′-TTGACAGCGACAAGAAGTGG-3′
	R: 5′-GTACATAGGGTACATGAGCG-3′
TNF-α	F: 5′-AGCCCATGTTGTAGCAAACC-3′
	R: 5′-TGAGGTACAGGCCCTCTGAT-3′
TGF-β	F: 5′-GTGGAAACCCACAACGAAAT-3′
	R: 5′-CACGTGCTGCTCTCACTTTTA-3′
GADPH	F: 5′-CGAGATCCCTCCAAAATCAA-3′
	R: 5′-TTCACACCCATGACGAACAT-3′

### Cell Invasion assay

Cell invasion assay was performed using 8-*µ*m pore size Transwell Biocoat Control inserts (Becton Dickinson) according to the manufacturer's instructions. Panc-1 cells were incubated for 48 hours. The cells were fixed with methanol and thereafter stained with Toluidine Blue (Merck). The number of transmembrane cells was counted.

### Statistical analysis

All reported values are expressed as mean+/−SD. For statistical analysis Mann-Whitney U test or a one-way ANOVA statistical test followed by Bonferroni's *t* test were used.

## Results

### Literature analysis using the CRAB tool and hypothesis generation

**Figure 1:**
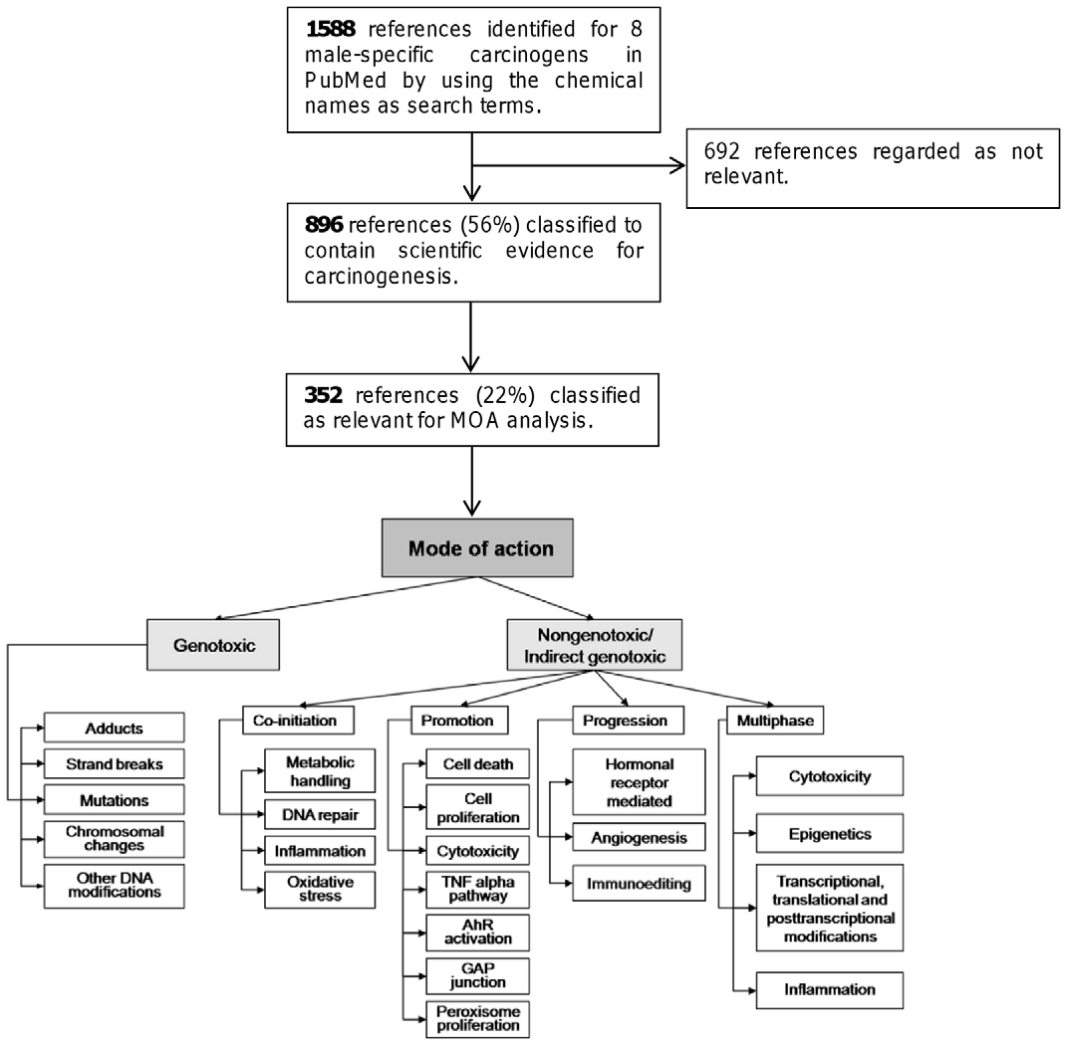
Flow chart of the tool used for classifying the abstracts.

The analysis of the NTP database revealed that eight chemicals induced tumors in exocrine pancreas (pancreatic acinar cell adenoma or carcinoma) in male rats only (Table 1). These NTP data showed that the eight chemicals had carcinogenic properties in male pancreas, but did not provide any information about mechanisms. To investigate whether the common tumor distribution reflected common toxicological effects among this group of chemicals we gathered the literature from PubMed via a search based on chemical names and retrieved 1588 abstracts (Table 1). We used the CRAB tool to analyse the abstracts [21]. The tool is illustrated in the flow chart shown in Figure 1, and classifies abstracts automatically according to a taxonomy for “mode of action” (MOA) for a given chemical.

1588 PubMed abstracts were thus identified (August 2011) and the number of abstracts per chemical ranged from 14 to 1028 abstracts (Table 1). The tool identified 352 of these abstracts as relevant for MOA classification and automatically classified 78% as irrelevant. This happened in two steps; in the first step 692 abstracts were excluded from further analysis due to lack of evidence for carcinogenicity, and in the second step 544 abstracts were excluded as irrelevant for MOA analysis (Figure 1). This reduced the reading load. Among the remaining 352 abstracts the tool performed the “genotoxic”/“non-genotoxic” classification. We found that the tool classified 39% of the 352 abstracts as “genotoxic” and 46% as “non-genotoxic”. This is in line with NTP results based on Salmonella testing which scored three as genotoxic and five as non-genotoxic (Table 1). We also analysed six well-known genotoxic chemicals and ten well-known non-genotoxic chemicals. As expected, for the genotoxic reference group the majority of abstracts (63%) were classified as “genotoxic”, while for the non-genotoxic reference group the majority were classified as “non-genotoxic” (76%). Thus the tool's analysis is consistent with the fact that the eight test chemicals are a mix of genotoxic or non-genotoxic chemicals.

We then analysed the sub-classes of MOAs (Figure 1). Table 3 shows sub-classes with 1% or more abstracts. For the genotoxic reference group the sub-classes called “adducts” and “mutations” gave the highest scores while for the non-genotoxic reference group the highest scoring sub-classes were “oxidative stress”, “cell death” and “cell proliferation” (Table 3). This is in line with previous experiments using the tool [21], [23]. The distribution for the eight chemicals causing exocrine pancreas tumors was more even. The 352 abstracts were distributed in both groups of sub-classes in the taxonomy (Table 3), and “mutations” and “cell proliferation” were common assignments. “Inflammation” was also a common assignment (10% versus 1% for the two other groups of chemicals; Table 3) and further analysis revealed that for five of the eight chemicals many articles concerning inflammatory effects were found. These results were informative as they are consistent with the generally accepted notion that inflammation is a prominent factor in pancreatic tumorigenesis [16], [27]. Guided by the results generated by the tool we hypothesized that many pancreatic carcinogens act by inducing or aggravating inflammation, and continued our investigation by experimental studies.

**Table 3:** Automatic classification of PubMed abstracts for genotoxic, non-genotoxic and male-specific pancreatic carcinogens.

MOA nodes	Genotoxic carcinogens (n = 6)	Non-genotoxic carcinogens (n = 10)	Male-specific pancreatic carcinogens (n = 8)
**Genotoxic MOA**	63%	10%	39%
**Nongenotoxic MOA**	34%	76%	46%
**Genotoxic sub-MOAs**			
Strand breaks	2%	1%	3%
Adducts	16%	1%	2%
Micronucleus	4%	1%	4%
Mutations	31%	3%	15%
**Nongenotoxic sub-MOAs**			
Oxidative stress	3%	6%	4%
Inflammation	1%	1%	10%
Cell proliferation	3%	9%	9%
Cell death	2%	11%	3%
Cytotoxicity	3%	3%	4%

Aggregated abstracts for the three categories of carcinogens (as specified in Materials and methods) were distributed by the CRAB tool in the taxonomy shown in Figure 1. Only nodes assigned 1% or more of the abstracts are shown.

### P2X7, calcium release, calcineurin induction and increased autotoxin expression in human Panc-1 cells

In order to focus experimental work on relevant targets we manually inspected the papers assigned to the inflammatory node and found that one of the eight chemicals, TDI, has previously been shown to affect intracellular Ca^2+^concentration [Ca^2+^] via purinergic P2X receptors [28], [29]. One of these receptors, P2X7, is over-expressed in malignant pancreatic tissues and in chronic pancreatitis [30]. Furthermore, P2X7 activation may result in lowered pH, a factor of importance in acute pancreatitis [31]. We thus tested the possibility that the pancreatic carcinogens induced changes in [Ca^2+^] and inflammatory proteins in human ductal Panc-1 cells.

**Figure 2:**
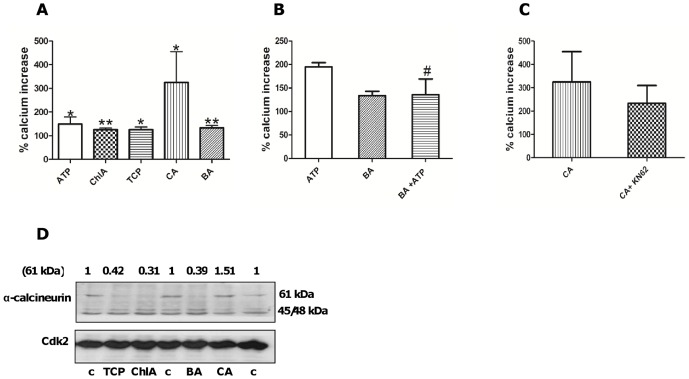
Male-specific pancreatic carcinogens increase Ca^2+^ levels and activate α-calcineurin. (**A**) Fura-2-loaded Panc-1 cells were treated with ATP (200 µM), ChlA (100 µM), TCP (1 mM), CA (50 µM) or BA (500 µM). *significantly different from controls (set to 100%) (*p<0.05, **p<0.01). (**B**) BA+ATP-treated cells were pretreated with BA and thereafter with ATP. #significantly different from ATP, p<0.05. (**C**) Cells were preincubated with KN62 (100 nM) for 10 min followed by CA (50 µM) treatment. (**D**) Cells were treated with chemicals (10 µM) for 15 min. Levels of α-calcineurin (45/48 kDa, active form and 61 kDa, inactive form) were analyzed by Western blotting. Three different controls (c) were used. In A, B and C results are presented as mean ± SD, n = 3.

In a first series of experiments we investigated the ability of BA, CA, ChlA and TCP to induce an increase in [Ca^2+^]. ATP, the natural P2X7 ligand, was used as positive control. All four chemicals significantly increased [Ca^2+^] in human Panc-1 ductal cells compared to control (Figure 2A). We also found that BA, CA and TCP affected the ATP response. Thus pre-treatment with BA (Figure 2B) prevented or attenuated the effect of ATP (added two minutes after BA). A similar effect was observed for CA and TCP (data not shown). KN62 is a selective P2X7 antagonist and we found that KN62 attenuated the effect of CA on [Ca^2+^] (Figure 2C).

Calcineurin is an inflammatory protein activated by [Ca^2+^]. It has been associated with pancreatitis [31], [32] and we investigated effects on calcineurin. Calcineurin activation involves cleavage of the 60 kDa protein to form a 45/48 kDa product. We found that calcineurin was activated after 15 minutes treatment by BA, ChlA and TCP, but not by CA (Figure 2D).

**Figure 3:**
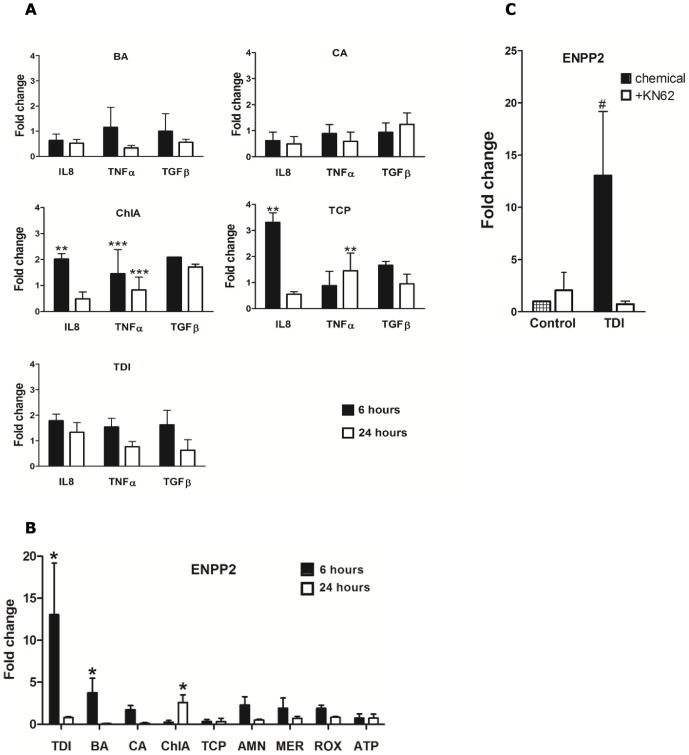
Male-specific carcinogens induce increased expression of inflammatory markers in Panc-1 cells. (**A**) Cells were treated with 10 µM BA, CA, ChlA, TCP or TDI for 6 or 24 hours. Samples were analyzed by Real Time RT-PCR using primers shown in Table 2. Results are presented as mean ± SD, n = 3 and control levels were set to one. (**B**) Cells were treated with 10 µM TDI, BA, CA, ChlA, TCP, AMN, MER, ROX or ATP for 6 or 24 hours. Samples were analyzed by Real Time RT-PCR using primers for *ENPP2* shown in Table 2 (**C**) mRNA levels for *ENPP2* in cells treated with 10 µM TDI for 6 hours in presence or absence of 100 nM KN62 (10 min pretreatment) detected by Real Time RT-PCR. Results are presented as mean ± SD, n = 3. *significantly different from controls (*p<0.05, **p<0.01, ***p<0.001, #p = 0.07).

Although we only tested four of the eight chemicals, these data suggested that they commonly affected intracellular [Ca^2+^] and activated calcineurin. We now studied the mRNA levels of four inflammatory genes (*IL8, TNFα, TGFβ* and *ENPP2*) which all have been connected to calcineurin activation, as e.g. *ENPP2* [33]. Panc-1 cells were exposed to BA, CA, ChlA, TCP and TDI for 6 or 24 hours. Real-time RT-PCR was performed and the results showed a complex response. Both *IL8* and *TNFα* were significantly induced by ChlA and TCP (Figure 3A). Interestingly, *ENPP2* was significantly induced by three chemicals (BA, ChlA and TDI) and TDI increased the level almost 15-fold (Figure 3B), so in further *ENPP2* analysis we included all 8 chemicals. We found that levels of *ENPP2* mRNA were significantly increased by BA, TDI (6 hours) and by ChlA (24 hours). We also noted a non-significant increase for AMN, MER and ROX. No apparent increase was observed for TCP or ATP (Figure 3B). Also shown in Figure 3C is the inhibitory effect of the P2X7 inhibitor KN62 on *ENPP2* mRNA levels induced by TDI (6 hours).

*ENPP2* encodes autotaxin (ATX), an intracellular and excreted lysophosphatase that has been associated with development of aggressive cancer types, including pancreatic cancer [34], [35], [36]. Furthermore, it has been suggested that a spliced variant of ATX is regulated by [Ca^2+^] [37], and data also indicate that P2X7 induces lysophosphatidic acid (LPA) production [38], [39]. We thus posed the question if a stimulated ATX expression could be a common cellular effect of chemicals inducing tumors in pancreas.

### Pancreatic carcinogens increased autotaxin (ATX) protein levels

**Figure 4:**
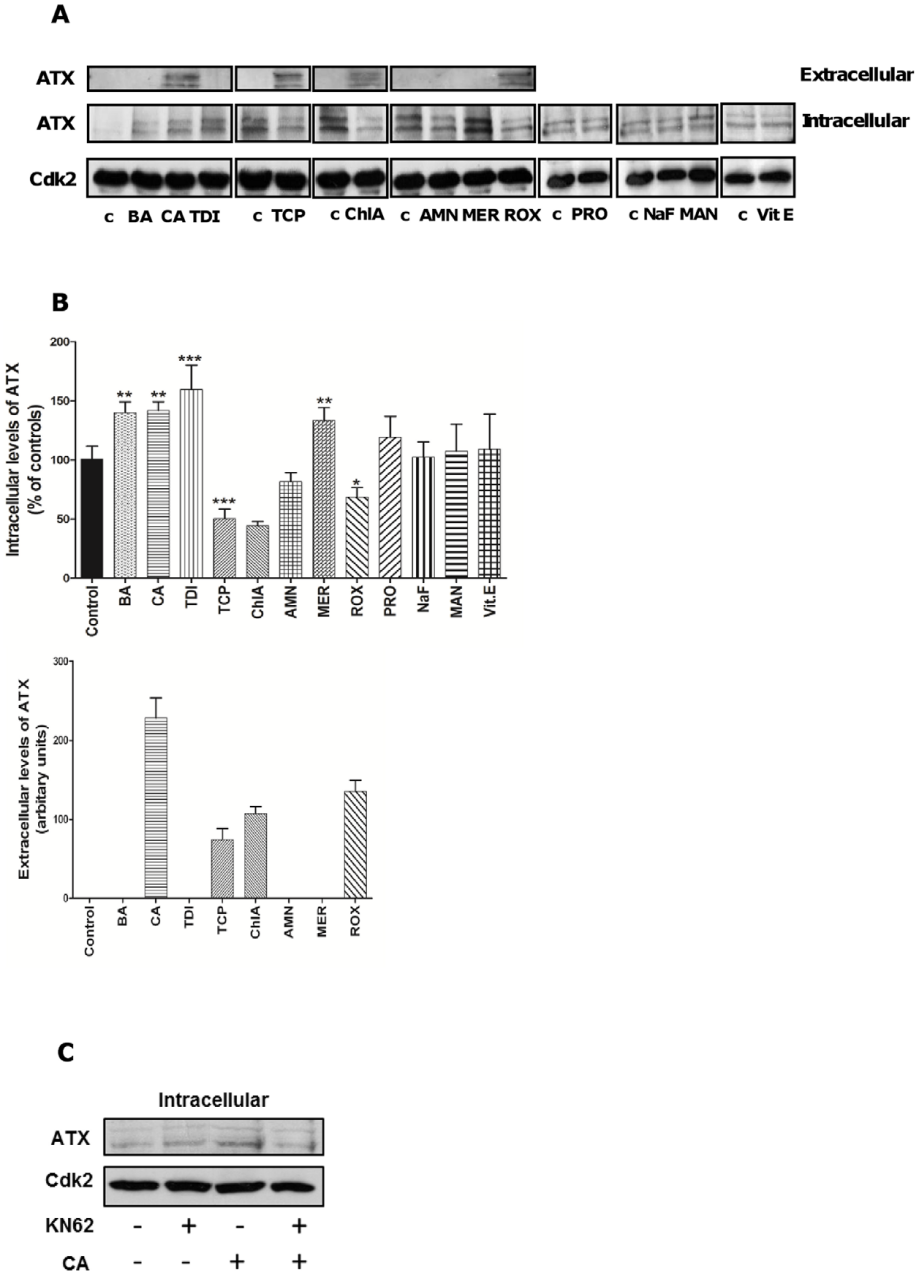
Male-specific pancreatic carcinogens induce ATX signaling in Panc-1. Cells were treated with 10 µM TCP, ChlA, BA, CA, TDI, AMN, MER, ROX, NaF, MAN, Vit E or PRO for 24 hours. (**A**) Representative Western blots of intracellular and extracellular ATX protein levels in Panc-1 cells. Different controls (c) were used (see Materials and Methods). (**B**) Densitometric analysis of intracellular and extracellular ATX levels in Panc-1 cells. Levels in untreated cells (c) were set to 100 (intracellular) or zero (extracellular). (**C**) Intracellular protein levels of ATX in cells treated with 10 µM CA for 24 hours in presence or absence of 100 nM KN62 (10 min pretreatment). Cdk2 was used as a loading control. Results are presented as mean ± SD, n = 3. *significantly different from controls (*p<0.05, **p<0.01, ***p<0.001).

We analysed intra- and extra-cellular ATX protein levels. As can be seen from Western blots in Figure 4A, we detected basal levels of ATX in Panc-1 cells. We also observed that one of the vehicles used, DMSO, decreased the basal cellular level (as shown in the first lane, Figure 4A). None of the control conditions affected extracellular levels of ATX.

As shown by the densitometric analysis BA, CA, TDI, and MER increased intracellular levels of ATX in Panc-1 cells and CA, ChlA, TCP, and ROX increased extracellular ATX (Figure 4B). Thus seven chemicals either increased intracellular (BA, CA, TDI, MER), extracellular (CA, ChlA, TCP, ROX) ATX levels or both (CA) (Figure 4B). One chemical (AMN) did not increase ATX levels in Panc-1 cells. We also tested four chemicals that did not induce pancreatic tumors in NTP bioassays, probenicid (PRO), mannitol (MAN), sodium fluoride (NaF) and *α*-tocopherol. None of these chemicals induced any change of intracellular ATX levels in Panc-1 cells (Figure 4A). In additional experiments we used two other human ductal adenocarcinoma cell lines, MIA PaCa-2 and Capan-2, and results obtained in the three cell lines are summarized in Table 4. As can be seen, all eight chemicals increased either intra- or extra-cellular levels of ATX in at least one cell line (Table 4). Figure 4C shows that KN62, the P2X7 inhibitor, alone did not affect intracellular ATX levels in Panc-1 cells. However KN62 attenuated the CA-induced ATX accumulation.

**Table 4:** Summary of results obtained in Panc-1, Mia-PaCa-2 and Capan-2 cells.

	BA	CA	TDI	TCP	ChlA	AMN	MER	ROX
**Panc-1 cells**								
ATX (intracellular)	**+**	**+**	**+**	**−**	**−**	**−**	**+**	**−**
ATX (extracellular)	**−**	**+**	**−**	**+**	**+**	**−**	**−**	**+**
MMP-9	**+**	**+**	**+**	**−**	**−**	**+**	**+**	**+**
Invasive assay	**+**	**+**	**−**	**+**	**+**	**+**	**+**	**+**
ATX + Invasive assay	**+**	**+**	**−**	**+**	**+**	**−**	**+**	**+**
**Mia-PaCa-2 cells**								
ATX (intracellular)	**−**	**−**	**−**	**−**	**−**	**+**	**−**	**−**
ATX (extracellular)	**+**	**+**	**+**	**−**	**−**	**+**	**−**	**+**
**Capan-2 cells**								
ATX (intracellular)	**+**	**+**	**+**	**−**	**−**	**+**	**+**	**+**
ATX (extracellular)	**+**	**+**	**+**	**+**	**−**	**+**	**+**	**−**

### Increased MMP-9 expression and invasive migration

**Figure 5:**
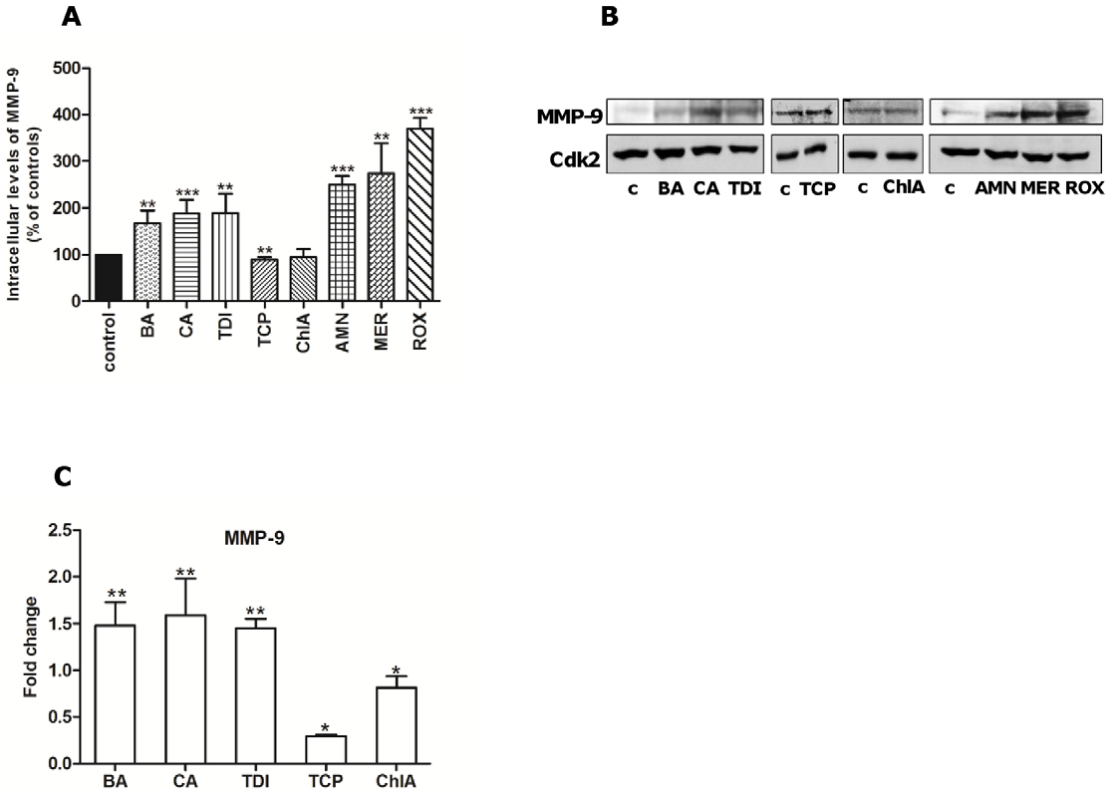
Male-specific pancreatic carcinogens induce MMP-9 expression in Panc-1 cells. Cells were treated with 10 µM TCP, ChlA, BA, CA, TDI, AMN, MER or ROX for 24 hours. (**A**) Densitometric analysis of intracellular MMP-9 levels. Level in untreated cells (control) was set to 100. (**B**) Representative Western blots of intracellular MMP-9 protein levels. (**C**) Cells were treated with 10 µM BA, CA, TDI, TCP or ChlA for 24 hours. Samples were analyzed by Real Time RT-PCR using primers shown in Table 2. Results are presented as mean ± SD, n = 3 and control levels were set to one. *significantly different from controls (*p<0.05, **p<0.01, ***p<0.001).

ATX has been shown to activate MMP-9 (Matrix Metalloprotease-9) through LPA receptor 1 [40] and may also be activated by P2X7 receptors [41]. MMP-9 is a collagenase belonging to the matrix metalloprotease group of proteins which degrade extracellular matrix during cancer cell invasion. MMP-9 has also been implicated in the progression of pancreatic tumors [42] and its expression is associated with pancreatic cancer and pancreatic tumor metastasis [43]. We investigated whether our eight test chemicals activated MMP-9. We found that BA, CA, TDI, AMN, MER, and ROX increased MMP-9 levels in Panc-1 cells (Figure 5). Figure 5A shows the densitometric analysis of three different experiments, and Figure 5B a representative Western blot. The chemicals which did not show the increase in MMP-9 levels were TCP and ChlA. Similar results were obtained in mRNA analysis (Figure 5C).

**Figure 6:**
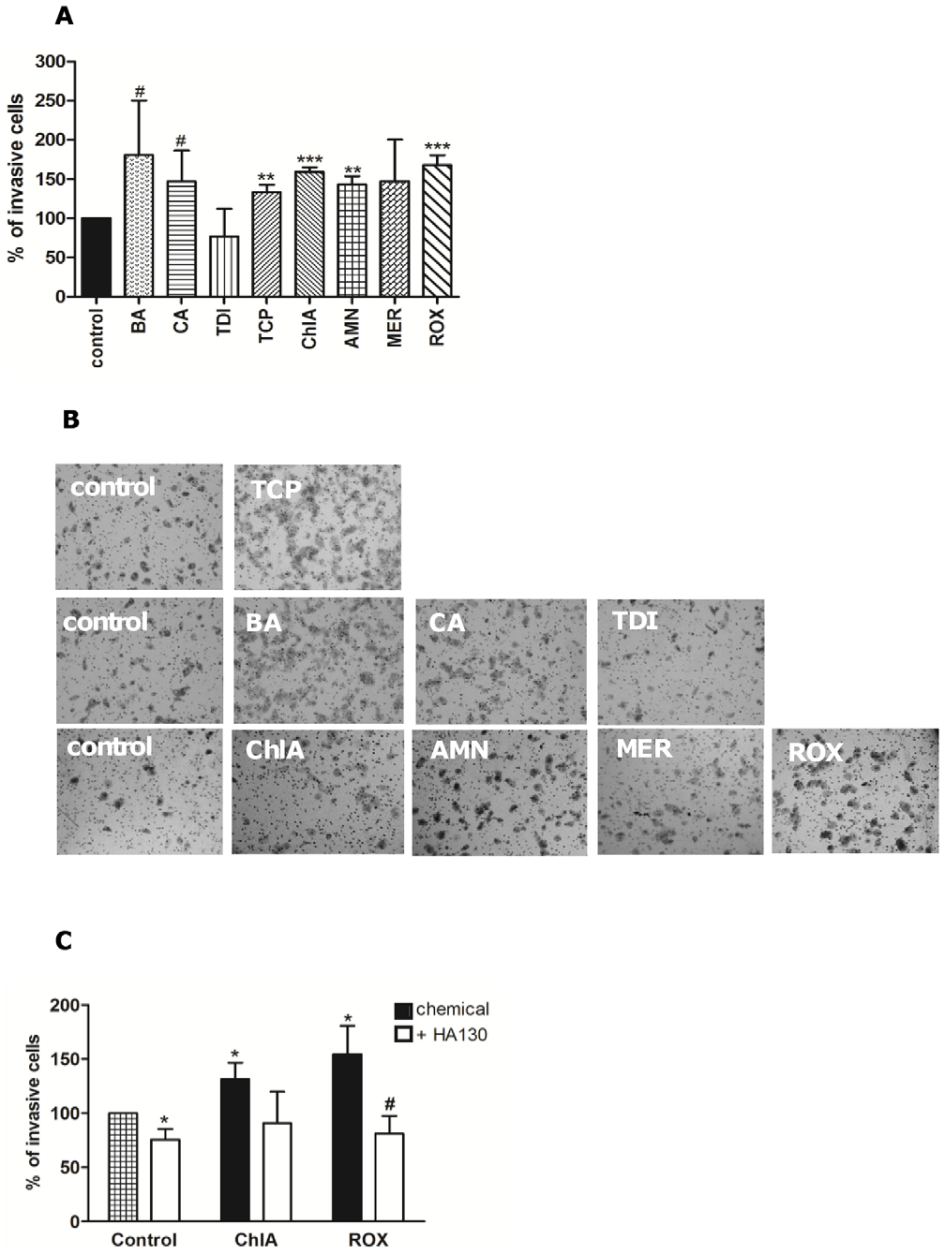
Male-specific pancreatic carcinogens increase invasive migration of Panc-1 cells. Cells were treated with 10 µM TCP, ChlA, BA, CA, TDI, AMN, MER or ROX for 24 hours. (**A**) Percentage of invasive cells after 48 hours. (**B**) Representative images of the invasive assay after 48 hours. (**C**) Cells treated with ChlA or ROX were pretreated with 330 nM HA130 (4 hours pretreatment). Results in 6A and 6C are presented as mean ± SD, n = 3. *significantly different from controls (^#^p≤0.06, *p<0.05, **p<0.01, ***p<0.001).

ATX and LPA have been shown to increase invasive growth [44], and next we investigated whether test chemicals increased invasiveness of the Panc-1 cells. As shown in Figure 6A and 6B all chemicals inducing male-specific tumors, except TDI, induced significant increase in the number of invasive cells. The invasive assay data are summarized in Table 4 and in Table S1, which also gives the evidence for overall carcinogenicity according to NTP. We also tested an ATX inhibitor, HA130. We tested it in combination with ROX and ChlA, which gave the most robust increases (Figure 6A). As shown in Figure 6C, HA130 inhibited both ROX- and ChlA-stimulated invasions in three experiments, although the effect on ChlA was not significant (p = 0.09). Interestingly, HA130 exhibited an inhibiting effect of its own, suggesting that basal ATX levels, as shown in Figure 4A, supported invasive growth in Panc-1 cells.

### Testosterone increases ATX levels

**Figure 7:**
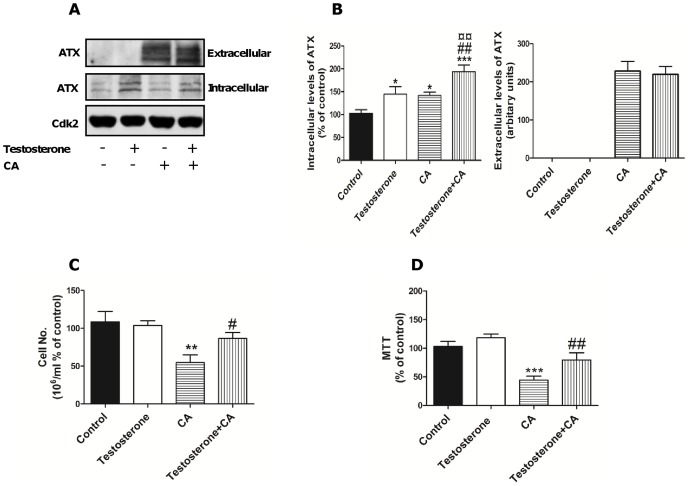
Testosterone increases CA-induced ATX signaling and prevent CA-induced toxicity in Panc-1 cells. Panel (**A**) and (**B**) show Western blots and densitometric analysis of extracellular and intracellular ATX levels. Cells were incubated with 1 nM testosterone for 24 hours and thereafter with 10 µM CA for additional 24 hours. (**C**) Cells were stained by trypan blue and counted under light microscope. Cells were preincubated with 1 nM testosterone for 24 hours and thereafter treated with 10 µM CA for additional 24 hours. (**D**) Cell numbers measured by MTT. Cells were preincubated with 1 nM testosterone for 24 hours and thereafter treated with 100 µM CA for additional 24 hours. Data was obtained from three independent experiments. Results are presented as mean ± SD. *significantly different from controls (*p<0.05, **p<0.01, ***p<0.001). #significantly different from CA alone (#p<0.05, ##p<0.01). ¤significantly different from testosterone alone (¤¤p<0.01).

In an effort to link the male dominance to ATX as a target for pancreatic carcinogens we investigated if testosterone affected ATX levels. Testosterone has been implicated in pancreatic cancer development in rats [20], and there are also data suggesting a role for testosterone in epithelial-to-mesenchymal transition (EMT) [45] and EMT including migration [46] in non-prostate tumors. As shown in Figure 7A, testosterone increased intracellular levels in Panc-1 cells but did not affect extracellular levels. To investigate possible interactions we tested if testosterone increased the effect of CA. CA was selected because it induced both intra- and extra-cellar levels of ATX in Panc-1 cells. As shown in Figure 7B the combined effect of CA and testosterone on intracellular ATX levels was additive. We also analysed the effects of CA and testosterone on cell growth. CA alone induced toxicity and decreased the number of cells as compared to controls. Testosterone partially prevented this effect (Figure 7C and D). These results suggest that CA has a toxic potential that may or may not be related to ATX induction, and that testosterone has the capacity to prevent this effect. Testosterone may thus not only increase intracellular ATX levels, but also prevent death of cancer cells. These data offer an explanation on how ATX might affect males more than females.

## Discussion

In this study we have examined chemicals inducing pancreatic tumors in the NTP database. We identified eight chemicals that induced exocrine pancreatic tumors in male rats only. We also found two chemicals that induced more pancreatic tumors in males than in females but no single chemical that induced exocrine pancreatic tumors in female rats only. These data extends the more than 20-year old observation [20], [47] that male rats are more susceptible than female rats to chemical induced tumors in exocrine pancreas. Our main finding is that the ATX-LPA-axis is targeted by all eight chemicals that caused male-specific rat pancreatic tumors. Our results and the way we selected our test chemicals suggests that ATX is a common target for chemical induced tumors in pancreas. Although testosterone also increased ATX levels, further studies are needed to fully understand the male dominance.

Guided by recent progress in the understanding of pancreatic carcinogenesis and by our text mining tool [21], [23] we searched for factors that might explain why the eight male-specific chemicals induced pancreatic tumors. We hypothesized that the induction of tumors in male pancreas reflects common toxicological effects and used the tool to search the literature for such effects. The tool automatically selected 352 abstracts and classified them according to the taxonomy capturing mechanistic information of relevance for carcinogenic activity. This analysis showed that chemicals inducing pancreatic tumors deviated from typical genotoxic and non-genotoxic carcinogens. Further analysis of the eight chemicals revealed that cell proliferation and inflammatory effects were frequently discussed in the literature. Subsequent manual reading of relevant articles, followed by experimental work, confirmed an involvement of inflammation. In short, the tool provided a rapid overview which suggested effects that these chemicals have in common, and by integrating automatic literature analysis with manual reading we saved time in the hypothesis generating processes. Perhaps most time was saved by the exclusion of 1236 abstracts (c.f. Figure 1) as non-relevant for the MOA analysis. Time-saving and hypothesis generation aspects of the tool in general have been discussed previously [21], [23].

We performed experimental studies employing human male pancreatic ductal cell lines. We started with a set of five chemicals (BA, CA, ChlA, TCP and TDI) and guided by literature data on TDI [28], [29], we found that ATX was frequently induced. Employing all eight pancreatic carcinogens and three pancreatic ductal cell lines we showed that all chemicals have the capacity to induce ATX. Although the ATX response varied between chemicals and cell lines - which might be explained by kinetic differences or by differences in mechanisms of induction - we conclude that ENPP2/ATX is induced by so far uncharacterized type(s) of stress induced by certain environmental chemicals. Except for a study on TCDD-induced ATX mRNA levels [33] we have found no reports on pollutants or toxic chemicals affecting ATX expression, so ATX induction as a response to environmental chemicals is a novel observation. Events possibly triggering ATX induction in our studies included a disturbed Ca^2+^ regulation and calcineurin activation, effects previously associated with the initiation of pancreatitis [31], [32]. An analogous signalling was proposed in the TCDD study [33], a dioxin which actually exhibits pancreatic carcinogenic activity [48], and which stimulated migration via calcineurin, NFATc1 and ATX induction in breast cancer cells [33]. We also documented a possible involvement of the P2X7 receptor, which is in line with human clinical data on pancreatitis and pancreatic cancer, indicating increased expression of P2X7 [30]. Interestingly, The Human Protein Atlas (www.proteinatlas.org) reports moderate ATX staining in exocrine pancreas and weak staining in islets of Langerhans. In nine pancreatic “adenocarcinomas” variable staining of ATX intensity was reported.

ATX is a recently characterized inflammatory marker, a lysophospholipase and motility factor. ATX regulates levels of serum and tissue lysophosphatidic acids (LPA) which activates six receptors, out of which at least four (LPAR1 - 4) are expressed in human pancreatic tissue [49]. The physiological regulation of ATX is not well characterized, but a recent study indicates a role for histone deacetylators [50], and our present data thus indicate induction by xenobiotics. ATX inhibition results in a rapid decrease in e.g. blood LPA levels [51], indicating a dominating influence of ATX on LPA levels.

ATX-LPA axis has been implicated in the carcinogenesis of exocrine pancreatic [34], [35], [36], [52] and some other inflammatory related tumors in humans [53], [54]. Perhaps the most direct evidence for an involvement in human pancreatic cancer is the observation that pancreatic cancer patients exhibited increased ATX serum levels [36]. Mechanistic studies indicate that ATX promotes tumor cell invasion via LPA, their receptors [55] and via MMP-9 [43]. In line with this, we showed induction of MMP-9 by BA, CA, TDI, AMN, MER, and ROX. ChlA and TCP did not exhibit this effect, but this might be explained by non-optimal timing of the experiments. The role of ATX in tumor cell invasion is in line with the observation that pancreatic cancer is invasive early during its development [48]. ATX may also stimulate epithelial-mesenchymal transition [56], and has been shown to promote metastasis [57], which is an additional characteristics of this tumor type [48]. Using the eight pancreatic tumor-inducing chemicals, we found a strong correlation between ATX inductions, activation of MMP-9 and increased invasive growth (Table 4). As human ductal pancreatic cancer cell lines were employed this suggests a possible mechanism for ATX-promoted pancreatic tumor development in humans.

Earlier studies indicate interactions between ATX and LPA and several onco- and suppressor genes of relevance for pancreatic cancer (see Table 5). These genes/proteins are potential up- or down-stream targets that may facilitate pancreatic tumor development. Perhaps of particular interest are interactions with CCK [58]. To our knowledge this aspect of ATX signalling has not been studied in pancreatic tissue or cells.

**Table 5:** Literature data on effects of the ATX-LPA axis on genes/proteins that have been implicated in exocrine pancreatic tumor development.

Gene/protein	Ref.*	ATX effects	Ref.**
Ras	[16]	ATX amplifies tumorigenesis of ras-transformed cells.	[76]
β-catenin	[16]	β-catenin regulates ATX expression and LPA activates β-catenin.	[77], [78]
p53	[16]	ATX-LPA down-regulates p53 and replicative senescence.	[79]
Mdm2	[16]	Mdm2 is up-regulated by ATX-LPA and may have the same effect as down-regulation of p53.	[80]
NF-kB	[81]	LPA activates NFkB.	[82]
Rac1	[83]	LPA activates Rac1.	[55]
Akt	[16]	LPA activates Akt.	[84]
TGFβ	[83]	LPA potentiates the effect of TGFβ.	[85]
CCK	[15]	LPA induces CCK expression.	[58]
Stat3	[86]	Stat3 mediates migration via ATX expression.	[87]

* Reference for each gene/protein describes its association to exocrine pancreatic cancer.

** References describe effects of the ATX-LPA-axis. CCK: cholecystokinin.

ATX is produced in both males and females and our data do not suggest a clear “male-specific” MOA. A role of testosterone in pancreatic cancer development has been indicated previously [20], [47], [59] and our finding that testosterone increases ATX levels suggests a mechanism that supports a role of the ATX-LPA-axis in pancreatic carcinogenesis. However, a negative effect of estrogen has also been shown [47], as well as a complex influence of female sex hormones on LPA receptors [60], [61]. Furthermore, ATX levels in women are even higher than in men [62], so additional studies to fully understand gender aspects are obviously needed.

From a risk assessment perspective, it has been claimed that rat pancreatic acinar tumors may not be indicative for human risk [63], [64]. Rats (and wild-type mice) usually develop acinar exocrine tumors in response to carcinogens, whereas acinar tumors are rare in humans. They are seen in less than 2% of human cases [2] and the dominating human exocrine cancer exhibit ductal differentiation [16], [63]. The sequence of events leading to ductal tumors and mutation spectra [65], [66], [67] also argue for a fundamental difference between ductal and acinar tumors. However, this does not exclude a role for ATX in both tumor types, and, as indicated in Table 5, there are several possible mechanisms. Furthermore, cerulein-induced chronic pancreatitis leads to ductal carcinoma in mice harboring mutated K-ras in acinar or centroacinar cells [15], and differentiated adult pancreatic cells exhibit great plasticity [68], [69]. For example, an acinar cell origin of the putative ductal tumor precursor lesions (PanINs) [16] have been indicated in transgenic mouse models [70], [71], [72], [73]. So an involvement of ATX in ductal pancreatic carcinoma development in humans seems possible, and if it e.g. can be shown that increased ATX levels in serum [36] reflect ductal pancreatic carcinoma development in humans, the widely held assumption that acinar rat tumors do not predict risk is challenged. Further studies on the role of ATX in rat and human pancreatic cancer are thus warranted. It can also be added that arguments used to disqualify [64] rat acinar tumors as relevant for human acinar tumors are weak and circumstantial [74], [75], and should be further studied.

In summary, this study shows that the eight chemicals inducing rat acinar pancreatic tumors stimulate ATX formation in human pancreatic cancer cell lines. MMP-9 activation and increased invasive growth were also common and implicates ATX mechanistically in human pancreatic tumor development. Several lines of evidence in the literature support a role for the ATX-LPA axis in human pancreatic tumor progression, including pancreatitis-like effects [58]. A possible causative role for ATX in pancreatic tumor progression should be further investigated with the aim to better understand carcinogenesis in pancreatic models and their possible relevance for humans. Such studies may also lead to the development of biomarkers for studying chemical risk factors in humans.

## Supporting Information

Table S1 **Results obtained in Panc-1 cells arranged according to number of positive evidence related to ATX activation, MMP-9 activation and invasive growth.**
(DOCX)
