# Supplementary material for: Exocrine Pancreatic Carcinogenesis and Autotaxin Expression
Source: PLoS One. 2012 Aug 29;7(8):e43209. doi: 10.1371/journal.pone.0043209 (PMC3430650; doi:10.1371/journal.pone.0043209)
Supplement: Table S1 — Results obtained in Panc-1 cells arranged according to number of positive evidence related to ATX activation, MMP-9 activation and invasive growth. (DOCX) [file pone.0043209.s001.docx]

**Table S1**. Results obtained in Panc-1 cells arranged according to number of positive evidence related to ATX activation, MMP-9 activation and invasive growth.

|  | **CA** | **MER** | **BA** | **ROX** | **TCP** | **ChlA** | **AMN** | **TDI** |
| --- | --- | --- | --- | --- | --- | --- | --- | --- |
| **Panc-1 cells** |  |  |  |  |  |  |  |  |
| ATX (intracellular) | **+** | **+** | **+** | **-** | **-** | **-** | **-** | **+** |
| ATX (extracellular) | **+** | **-** | **-** | **+** | **+** | **+** | **-** | **-** |
| MMP-9 | **+** | **+** | **+** | **+** | **-** | **-** | **+** | **+** |
| Invasive assay | **+** | **+** | **+** | **+** | **+** | **+** | **+** | **-** |
| Number of positive parameters | 4 | 3 | 3 | 3 | 2 | 2 | 2 | 2 |
| Evidence levels according to NTP^a^ | **CE** | **SE** | **EE** | **EE** | **CE** | **CE** | **SE** | **CE** |

^a^overall evaluation of carcinogenicity by National Toxicology Program (NTP). It should be noted this evaluation is based on all tumors, not just pancreatic tumors. CE: clear evidence, SE: some evidence, EE: equivocal evidence.
